# USF1 Transcriptionally Regulates UGT1A3 and Promotes Lung Adenocarcinoma Progression by Regulating Neurotrophin Signaling Pathway

**DOI:** 10.3389/fmolb.2022.758968

**Published:** 2022-01-27

**Authors:** Yu Wang, Yun-Xia Zhao, Xiang-Wei Zhang, Yuan-Zhu Jiang, Wei Ma, Lin Zhang, Wei Dong

**Affiliations:** ^1^ Department of Oncology, Shandong Provincial Hospital Affiliated to Shandong First Medical University, Jinan, China; ^2^ Department of Neurology, Shandong Provinacial Hospital Affiliated to Shandong First Medical University, Jinan, China; ^3^ Department of Thoracic Surgery, Shandong Provincial Hospital Affiliated to Shandong First Medical University, Jinan, China

**Keywords:** upstream transcription factor 1, lung adenocarcinoma, UGT1A3, neurotrophin signaling pathway, the Cancer Genome Atlas

## Abstract

**Background:** Lung cancer remains the leading cause of oncological death. There is an urgent need to discover new molecular targets and to develop new treatments. Our previous study showed that one of the UDP-glucuronosyltransferases (UGTs) family, UGT1A3, is an important prognostic factor for lung adenocarcinoma (LUAD), inhibiting UGT1A3 could significantly improve the efficacy of anti-tumor drugs. In this study, we aimed to explore the upstream transcriptional factor (USF1) of UGT1A3 and its way of playing a role in LUAD.

**Methods:** The UGT1A3 promoter region was analyzed and dual-luciferase assay was involved to explore whether USF1 could bind to this region, and the possible regulation effects of USF1 to UGT1A3 was indicated by siRNA and recovery experiment. Then, the Cancer Genome Atlas database was used to analyze USF1 clinical features. The expression level of USF1 was detected by immunohistochemical assay and Western blotting. Cellular viability, proliferation, migration and invasion potential were also investigated. Meanwhile, the effect of USF1 in LUAD progression was detected in a mouse model. The downstream signaling pathway was analyzed by bioinformatic analysis and the expression of all related proteins was detected.

**Results:** UGT1A3 was transcriptionally regulated by USF1, which was highly expressed in all investigated samples including patients’ tissues, studied cells lines, and mouse models. The knockdown of USF1 inhibited cells viability, proliferation, migration and invasion, and reduced the tumor volume. Moreover, USF1 promoted the progress of LUAD by regulating the neurotrophin signaling pathway.

**Conclusion:** As an important transcriptional regulator of UGT1A3, USF1 was highly expressed in LUAD and promoted LUAD progression by regulating the neurotrophin signaling pathway. These findings provide a new theoretical data that could serve as a good foundation for the treatment of LUAD.

## Introduction

Lung cancer is the most common malignant tumor and the main cause of cancer related death worldwide. LUAD is a type of non-small-cell lung carcinoma (NSCLC), which accounts for about 80%–90% of NSCLC worldwide (Siegel et al., 2020). Approximately 70% of LUAD patients have local progression or metastasis at the time of diagnosis (Molina et al., 2008). Most patients were first diagnosed as locally advanced or too advanced for surgical treatment. The average 5-year survival rate of LUAD was less than 20% (Imielinski et al., 2012). Although various treatment methods including surgery, radiotherapy and chemotherapy, have been improved in recent years, the results are yet pessimistic (Vijayvergia and Mehra, 2014). Targeted therapy for patients with epidermal growth factor receptor (EGFR) and other mutations had made good progress. However, drug resistance remained a problem difficult to avoid. Even if the third-generation tyrosine kinase inhibitor (TKI) treatment was effective, drug resistance would occur soon (Andrews Wright and Goss, 2019). Therefore, there is an urgent need to develop effective markers to reveal the biological characteristics of LUAD, and to provide effective treatment accordingly.

UDP-glucuronosyltransferases (UGTs) are an important class of phase II drug metabolizing enzymes (Strassburg et al., 2000). UGTs catalyze the glucuronidation of many important endogenous compounds such as bilirubin, bile acids, thyroid and steroid hormones, as well as a large number of carcinogenic exogenous substrates (Yilmaz et al., 2015). UGT1A is one of the three subfamilies (UGT1A, UGT2A and UGT2B) (Hanioka et al., 2012) and was recently confirmed to promote drug resistance in some cancers (Scherer et al., 2014; Liu et al., 2015). Our previous results demonstrated that UGT1A3 was highly expressed in LUAD and associated with poor prognosis, inhibiting UGT1A3 showed significant anti-cancer effects by enhancing the activity of anti-tumor drugs (Wang et al., 2019). However, the specific mechanism and transcriptional regulation of UGT1A3 overexpression in LUAD remained unclear.

Human upstream transcription factor 1 (USF1), a member of the helix-loop-helix leucine zipper family, is located in the region q22.3 of chromosome 1. It contains 11 exons and has a total length of 5,734 kb. USF1 is a ubiquitously expressed transcription factor that regulates gene transcription by binding to the E-box motif of target genes (Ikeda et al., 2014). USF1 regulates genes involved in lipid and sugar metabolism (Kristiansson et al., 2008; Plaisier et al., 2009). Furthermore, it was found to be a potential marker of patient’s susceptibility to gastric carcinogenesis (Costa et al., 2020) and to promote glioma cell invasion and migration (Wang et al., 2020). Previous studies show that USF1-induced overexpression of the long noncoding RNA WDFY3-AS2 promoted LUAD progression (Ren et al., 2020). To date, the specific molecular mechanism by which USF1 exerts its effects on LUAD are yet incomplete and need further study.

In the current study, we found that USF1 was an important upstream transcriptional regulator of UGT1A3, the upregulated expression of USF1 was associated with advanced stage, nodal metastases, and poor survival rate in LUAD as UGT1A3. While its knockdown lead to inhibition of tumor cells’ proliferation, migration and invasion potentials *via* downregulation of UGT1A3. We further revealed that USF1 promoted lung adenocarcinoma progression through the neural signaling pathway (P75NTR, RIPK2, IRAK1, TRAF5 and IKKβ axis). As UGT1A3 was closely related to tumor drug resistance, discovery of transcriptional relationship between USF1 and UGT1A3 may provide a new idea for the study of drug resistance and therapy, and USF1 could serve as a potential therapeutic target for LUAD.

## Materials and Methods

### Cell Culture

Human NSCLC cell lines A549, H1299, PC-9, and H1975 (Chinese Academy of Sciences Cell Bank, Shanghai, China) were cultured in RPMI-1640 medium containing 0.1% double antibiotics (50 U/ml penicillin and streptomycin) and 10% fetal bovine serum, and were incubated at 37°C.

### Tissue Samples

Samples of lung adenocarcinoma tissues (2 cm away from the tumour margin) and adjacent normal tissues were taken from 30 patients who underwent surgical resection at the Shandong Provincial Hospital Affiliated to Shandong First Medical University (Jinan, China). The inclusion criteria were as follows: 1) None of the patients received neoadjuvant therapy; 2) all specimens were confirmed as LUAD by HE staining.; and 3) diagnosis was confirmed by two independent pathologists. The exclusion criteria were as follows: 1) Lung cancer cases with unconfirmed pathology; 2) lung cancer cases with incomplete data records; and 3) patients receiving chemotherapy and radiotherapy prior to the surgery. Informed written consent for scientific use of the biological material was obtained from each patient, and this study was approved by the Ethics Committee of Cancer Institute of Shandong Province. All experiments were carried out in accordance with the Declaration of Helsinki.

### Transfection

SiRNA1, siRNA2, and siRNA3 specifically targeting USF1 were synthesized and purified by RiboBio (Guangzhou, China). The USF1 specific short hairpin RNAs (shRNAs) and control shRNA were synthesized and produced by GenePharma (Shanghai, China). SiRNA specifically targeting UGT1A3 was synthesized and purified by RiboBio (Guangzhou, China). IRAK1 was cloned into pCDNA3.1 vector and an HA tag. Ttransfection was performed using Lipofectamine 2000 (Invitrogen, Carlsbad, CA, United States). Transfection efficiency was proved by Western blotting (WB).

### Western Blotting

Protein samples were extracted and quantified by RIPA buffer (Biovision, American) and protein concentration detection kit (Guangzhou Yingdante Science &Technology Co., Ltd., Guangzhou, China). Then, proteins were separated by 10% SDS-PAGE and transferred to polyvinylidene fluoride (PVDF) membranes (Millipore corp., Billerica, MA, United States). Non-specific sites were blocked with 5% milk powder diluted in TBS with .05% Tween 20 (TBST). After that, membranes were incubated overnight at 4°C with rabbit polyclonal antibody anti-USF1 (abs115735, 1:500 dilution, Absin, Shanghai, China), rabbit UGT1A3 polyclonal antibody (H00054659-A01, 1:500 dilution, Abnova, Wuhan, China), and rabbit P75 neurotrophic factor receptor (P75NTR) polyclonal antibody (TA328682, 1:200 dilution, OriGene Technologies, Rockville, United States), rabbit receptor interacting serine/threonine kinase 2 (RIPK2) polyclonal antibody (abs130017, 1:500 dilution, Absin, Shanghai, China), rabbit interleukin-1 receptor-associated kinase 1 (IRAK1) polyclonal antibody (AF7290, 1:500 dilution, Beyotime Biotechnology, Shanghai, China), rabbit ikappaB kinase β (IKKβ) polyclonal antibody (AF7200, 1:300 dilution, Beyotime Biotechnology, Shanghai, China), while the rabbit anti-GAPDH polyclonal antibody (1:500, Cell Signaling Technology, Inc.; Shangahi, China) was used as a control. After washing of the membranes repeatedly with TBST, they were incubated with horseradish peroxidase (HRP)-conjugated goat anti-rabbit IgG (#BHR101, 1:5,000, Beijing Bersee Technology Co., Ltd., Beijing, China). Finally, the Western blots were assessed by enhanced chemiluminescence. The expression of the relative protein levels were quantified by densitometry using the Quantity One software (Bio-Rad, Hercules, CA, United States). Relative protein expression levels were normalized to GAPDH.

### Immunohistochemistry Assay

Tissue sections were paraffin-embedded and immunostained with an antibody against USF1 (ab180717, Abcam). The tissue specimens were routine dewaxed. EDTA (pH 8.0) was used for microwave repair. After that, the endogenous peroxidase was blocked by adding 3% hydrogen peroxide in distilled water after natural cooling, incubated at room temperature for 10 min, and rinsed three times with phosphate buffer (PBS) for 2 min. The specimens were covered with 1:100 diluted MAGE-A3 monoclonal antibody, incubated for 1 h at 37°C, and rinsed three times with PBS for 5 min each. HRP-labeled secondary antibody was added to cover the specimen, incubated for 20 min at 37°C, and rinsed with PBS. The HRP-labeled secondary antibody was added to cover the specimen and was incubated for 20 min at 37°C. Three times rinsing with PBS followed for 5 min each. Diaminobezidin (DAB) was added to control the color development under the microscope. Rinsing thoroughly with tap water, re-stained with hematoxylin, dehydrated, and observed under the microscope after the tablets were sealed.

### Cell Counting Kit-8 (CCK-8)

Firstly, log phase cells were seeded in 96-well plate and incubated at 37°C with 5% CO_2_. Then, 10 μl of CCK-8 solution was added to each well and incubation for 1–4 h followed. Finally, the 96-well plate was placed on the enzyme-linked immunoassay instrument, and then measured the absorbance at 450 nm.

### Colony Forming Assay

Cells were inoculated into a six-well plate (500 cells in each well), the medium was changed every 2 days. After 2 weeks, the culture medium was discarded, and cells were fixed with 10% formaldehyde, stained with crystal violet for 15 min. Then, the staining solution was discarded and cells were washed thoroughly with PBS. Finally, the growth status was observed under the microscope (Leica Microsystems, Wetzlar, Hesse, Germany).

### Wound Healing Assay

Cells were inoculated into a six-well plate (2 × 10^5^ cells per well). Twenty-four hours post-incubation, the cells were scratched with a pipette tip and were washed three times with PBS to remove the scratched cells. The remaining monolayer was then incubated at 37°C with 5% CO_2_ with serum-free medium, and at the time points 0 and 48 h photos were taken to measure the distance, i.e., the wound healing potential.

### Transwell Migration Assay

30 μl low-concentration matrix glue (BD Matrigel) diluted matrix glue was applied to each transwell chamber prior to cell inoculation. The transwell plate was then placed in the cell incubator for 4 h and cells (1.333 × 10^5^/ml) were inoculated after the matrix gel solidified. After inoculation, the cells were inoculated and placed into a cell incubator. At 24 h, the compartment was taken and after staining was photographed (Carl ZEISS, Jena, German).

For the migration assay, logarithmic phase cells were digested and inoculated to the bottom of the transwell chamber (1 × 10^5^). The cells were then cultured for 24–48 h, the transwell chamber was taken out and the cells in the chamber and the remaining matrigel glue were wiped with a cotton swab, and washed three times with PBS. After fixation and staining with polyoxymethylene and crystal violet, microscopic observations followed (Carl ZEISS, Jena, German) for data quantitation.

### Mouse Model and Transfection

C57BL/6 mice were purchased from Jinan Xingkang Biotechnology Co., Ltd. (Jinan, China). The animal house was maintained at a temperature of 22 ± 2°C with relative humidity of 50% ± 15% and 12 h dark/light cycle. Xenograft tumors were established by subcutaneous injection of H1299 cells into 6-week-old male C57BL/6 mice. Sh-USF1 group mice were transfected with sh-USF1 tail-vein injection. The tumor was monitored by Vernier caliper while the tumor volume was indicated by a × b^2^/2 (a for long diameter; b for short diameter) every 5 days after injected 10 days. After 30 days, mice were anesthetized with 1% pentobarbital sodium (35 mg/kg, Dainippon Sumitomo Pharma) and fixed on the plate. The tumors were then removed, weighed and analyzed after the mice were euthanized by slow release of carbon dioxide in a box. All experimental procedures were approved by the Ethics and Scientific Committees of our institution (No. 2017440) performed following the Guide for the Care and Use of Laboratory Animals.

### Dual-Luciferase Reporter Assay

The sequences of the wild type or mutant UGT1A3 promoter including USF1 binding sites were sub-cloned into the pMIR-luciferase reporter construct and each construct was co-transfected with USF1 into the cells using Lipofectamine3000 (Invitrogen, United States). The sequence length of wild type UGT1A3 promoter is 2000bp (Chr2: 233727127–233729126). The promoter sequence was synthesized by Genscript Biotech Corporation. The wildtype and mutated UGT1A3 promoter were synthesized separately. The wild-type and mutant UGT1A3 promoter vectors are the same, and the restriction site is KpnI/XhoI. After the cells were cultured at 37°C for 48 h, the cells were evaluated by a Dual-Luciferase Reporter Assay System following to the manufacturer’s instructions. The luminescence values of cells in every group were detected using Renilla luminescence activity as an internal reference.

### Data Sources and Bioinformatics Analysis

The Cancer Genome Atlas (TCGA) data were downloaded at the website of the University of California Santa Cruz (UCSC) cancer browser (http://genome-cancer.ucsc.edu). The potential effect of USF1 on the post-progression survival (PPS) rates of patients with Lung cancer was analyzed using the Kaplan-Meier method with Kaplan-Meier Plotter web tools (https://kmplot.com/analysis/) in “Lung cancer” panel (Győrffy et al., 2013; Győrffy, 2021). Difference in gene expression was analyzed by DNA micro-assay and Venn. We used the DEseq of R software to analyze the difference in gene expression. KEGG analysis was performed on the common differentially expressed genes (DEGs) of each dataset using R software. The String11.0 database (https://stringGdb.org/) was used to analyze the interactions of common DEGs encoded proteins.

### Statistical Analysis

Data in the present study were presented as means ± standard deviation (SD). Statistical analysis was performed using SPSS 22.0 (Chicago, IL, United States) and R software. One-way ANOVA or two-tailed Student’s t-test were used for comparisons between groups. Survival analysis was performed using Kaplan-Meier method. The relationship between the variables and patient’s survival status was performed by Multivariate Cox proportional hazards method. *p* < .05 was considered statistically significant.

## Results

### USF1 Transcriptionally Regulated UGT1A3 Expression to Promote the Growth and Migration of LUAD Cells

**FIGURE 1:**
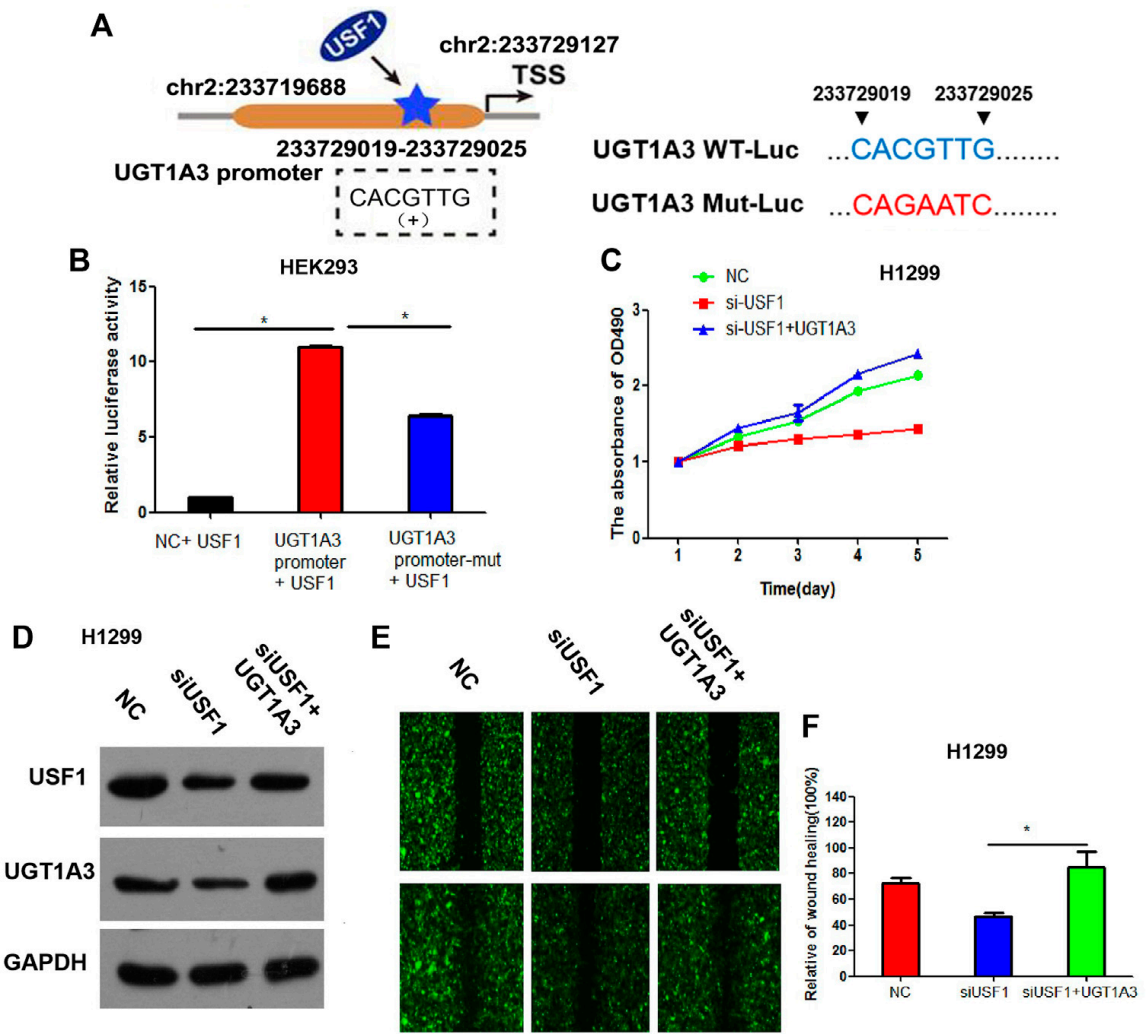
UGT1A3 expression was transcriptionally regulated by USF1. **(A)** A schematic representation of the location of the motif bound by USF1 at UGT1A3 promoter region. The UGT1A3 promoter region is located at chr2: 233719688–233729126. The USF1 site coordinates is chr2: 233729019–233729025. The coordinate position of TSS is chr2: 233729127. The positions of the UGT1A3 promoter and USF1 site are indicated according to human genome assembly GRCH38/hg38. **(B)** The relationship of USF1 and UGT1A3 was studied by Dual-luciferase reporter assay; **(C)** Cell viability was detected by CCK-8 assay in H1299 cell; **(D)** Transfection efficiency was determined by WB in H1299 cell; **(E,F)** Cell migration was detected by wound healing assay in H1299 cell. (si-USF1+UGT1A3 group stood for cells transfected with si-USF1 and pCDNA3.1-UGT1A3; *p < .05 means statistically significant difference when compared with NC group).

By analyzing the promoter sequences in Jaspar (http://jaspardev.genereg.net/) online tools, we found that the USF1 can bind to the promoter region sequence of UGT1A3 (CACGTTG, chr2:233729019–233729025) (Figure 1A).

To confirm whether USF1 can directly bind with the promoter of UGT1A3, luciferase reporter assay was performed. Results in Figure 1B showed that, relative luciferase activity was significantly increased in cells transfected with UGT1A3 promoter and USF1 overexpression vectors, as compared with the cells transfected with UGT1A3 promoter-mutant vectors (*p* < .05), suggesting USF1 was directly bound with the promoter of UGT1A3. Further study showed that, silence of USF1 using the specific targeted siRNAs significantly inhibited the viability of H1299 cells (Figure 1C). However, the viability loss induced by USF1 silence in H1299 cells was reversed by transfection of cells with UGT1A3 overexpression plasmids. Silence of USF1 remarkably downregulated the expression of USF1 and UGT1A3, while overexpression of UGT1A3 increased USF1 and UGT1A3 expression (Figure 1D). Additionally, silence of USF1 significantly inhibited the migration of H1299 cells, and its effects on cell migration were reversed by UGT1A3 overexpression (*p* < .05, Figures 1E,F). All these data suggested that USF1 transcriptionally regulated UGT1A3 expression *via* directly binding to its promoter, and thereby promoted the viability and migration of LUAD cells.

### Association Between USF1 Expression and Clinical Pathologic Characteristics of Patients With LUAD

**FIGURE 2:**
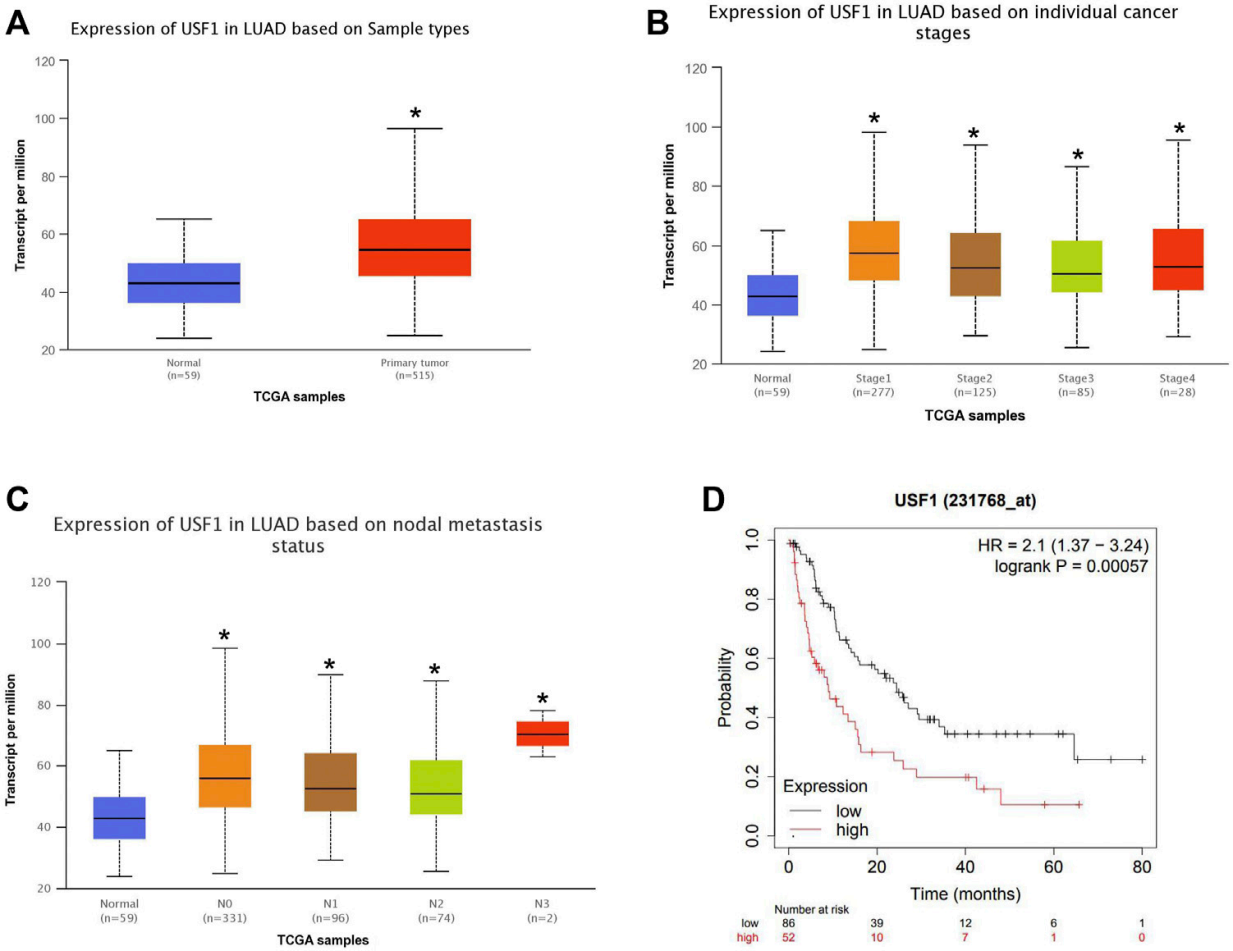
Relationship between the USF1 expression and patients’ clinical data in the TCGA database. **(A)** USF1 expression data in LUAD and normal tissues as reported in TCGA; **(B)** USF1 expression data in the category “Clinical stage” in TCGA; **(C)** USF1 expression data in the category “Nodal metastasis status” as reported in TCGA; **(D)** USF1 expression data in regard to the post-progression survival (PPS) rates analyzed by Kaplan-Meier Plotter web tools (https://kmplot.com/analysis/).

TCGA dataset was used to analyze the expression of USF1 in LUAD and its association with clinical pathologic characteristics of patients with LUAD. As indicated in Figure 2A, USF1 expression was higher in primary tumors (*n* = 515) than that in normal controls (*n* = 59) (*p* < .05). The expression of USF1 was successively increased in the LUAD samples of Stage 1–4 (*p* < .05, Figure 2B). The expression of USF1 was increased in the nodal metastases status especially in nodal metastases 3 (*p* < .05, Figure 2C). Moreover, Kaplan-Meier analysis based on the LUAD samples from the TCGA database showed a lower survival rate in patients with high level of USF1 as compared with those with low level of USF1 (*p* < .05, Figure 2D). These data showed that USF1 was highly expressed in LUAD, and the highly expressed USF1 has significant association with tumor stage, nodal metastases, and poor survival rate of patients with LUAD.

### USF1 Was Highly Expressed in LUAD Tissues and Cell Lines

**FIGURE 3:**
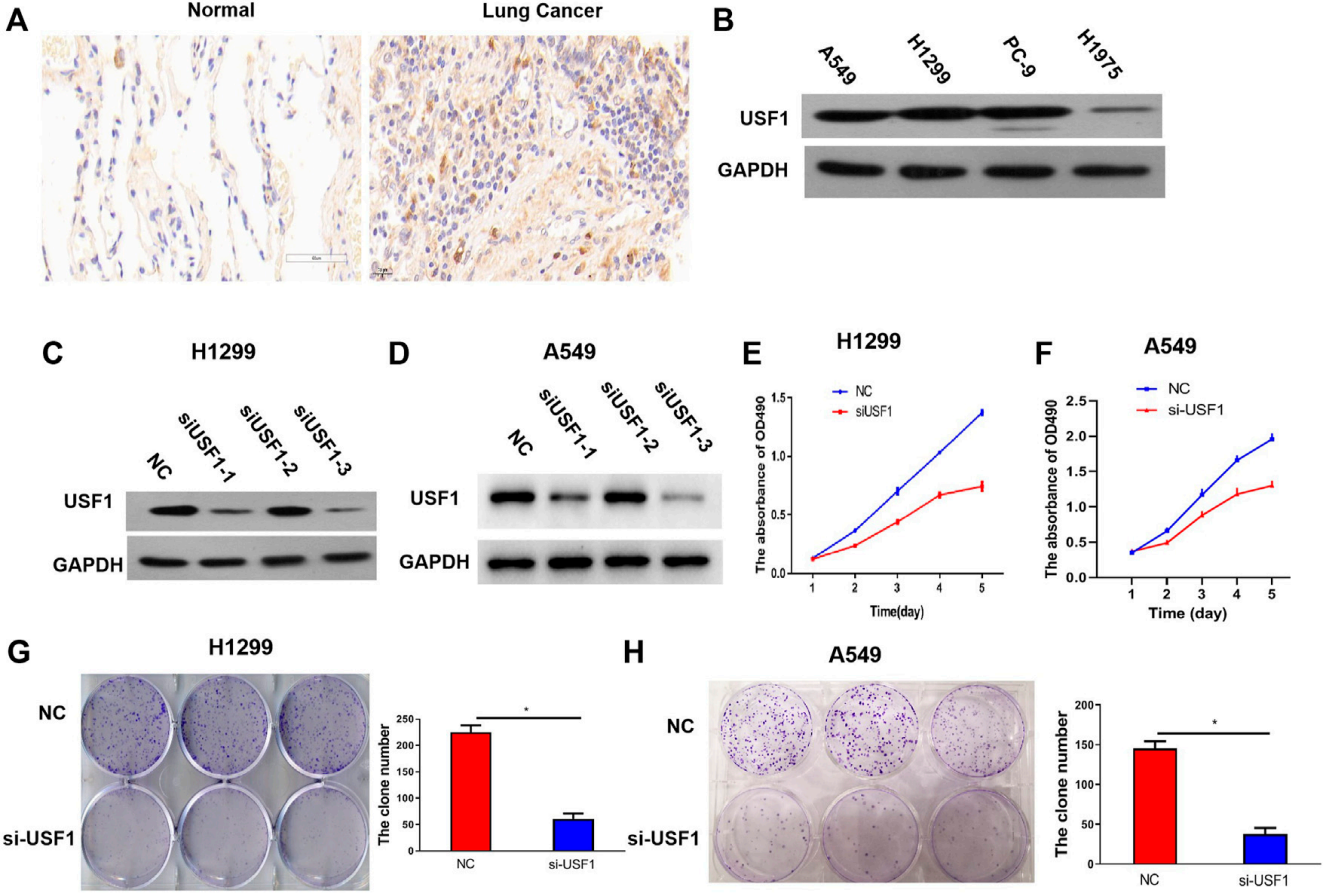
Protein expression and function investigation of USF1 in LUAD. **(A)** The USF1 protein expression in the studied tissues were determined by immunohistochemical assay; **(B)** The protein expression of USF1 in cells were determined by WB; **(C,D)** Transfection efficiency was proved by WB in H1299 and A549 cells; **(E,F)** Cells viability was detected by CCK-8 assay in H1299 and A549 cells; **(G,H)** cells proliferation potential was detected by the colony forming assay in H1299 and A549 cells. (NC group, cells were transfected with si-NC; si-USF1 group cells were transfected with si-USF1; *p < .05 means statistically significant difference when compared with NC group).

To further confirm the expression of USF1 in LUAD, 30 pairs of tumor and adjacent normal tissues were collected from patients with LUAD and the expression of USF1 was analyzed by immunohistochemical assays. As compared with adjacent normal tissues, USF1 was highly expressed in tumor tissues (Figure 3A). Western blotting analysis indicated that USF1 was frequently high expressed in several commonly used LUAD cell lines, including A549, H1299, and PC-9 (Figure 3B). To further reveal the effects of USF1 on LUAD cells, expression of USF1 in H1299 and A549 cells were silenced by transfection with specific targeted siRNAs (si-USF1-1, si-USF1-2 and si-USF1-3). As results shown in Figures 3C,D, protein levels of USF1 were remarkably decreased by siRNAs. Si-USF1-3 was selected for use in the following experiments, because it resulted in a lowest level of USF1 than other two siRNAs. Further study demonstrated that, the viability of H1299 and A549 cells was repressed by transfection of cells with USF1 siRNA as compared with NC (Figures 3E,F). In addition, the colony formation of H1299 and A549 cells was inhibited by transfection of cells with USF1 siRNA as compared with NC (*p* < .05, Figures 3G,H). There data confirmed the high expression of USF1 in LUAD and its silence was capable of inhibiting LUAD cell proliferation.

### USF1 Knockdown Inhibited the Migration and Invasion of LUAD Cells

**FIGURE 4:**
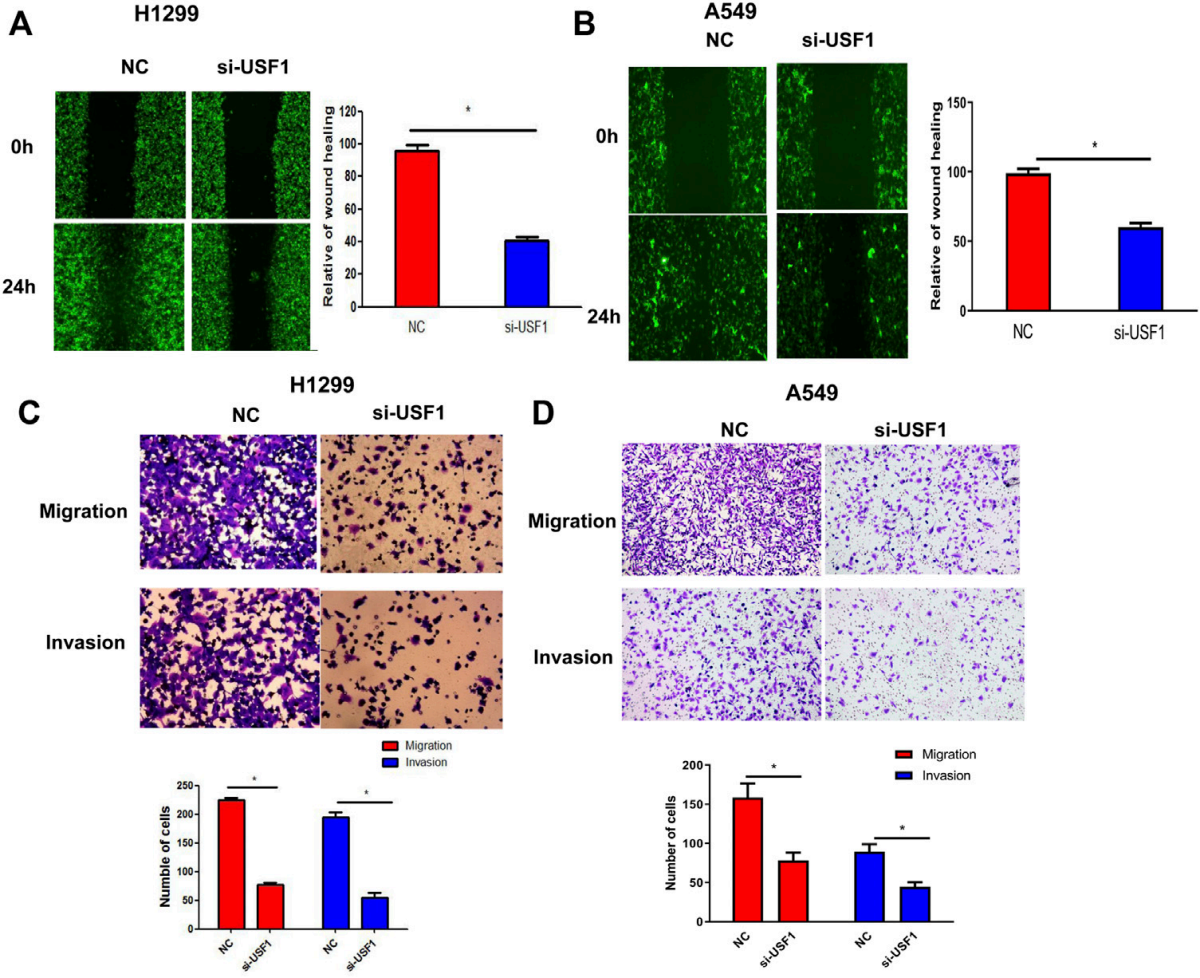
Investigation of USF1 on LUAD cells migration and invasion potentials. **(A,B)** Cells migratory potential was detected by wound healing assay in H1299 and A549 cells; **(C,D)** Cellular migratory and invasion potentials were detected by the transwell assay in H1299 and A549 cells. (*p < .05 means statistically significant difference when compared with NC group).

Cellular migration and invasion were determined by the wound healing and transwell migration assays. As shown in Figures 4A,B, the wound healing area in H1299 and A549 cells transfected with USF1 siRNA was significantly decreased in comparison with the NC group (*p* < .05). Transwell assay results indicated that, migrated and invaded cell number was significantly decreased in H1299 and A549 cells transfected with USF1 siRNA as compared with NC (*p* < .05, Figures 4C,D). All these results indicated that the silence of USF1 inhibited LUAD cell migration and invasion.

### USF1 Knockdown Inhibited Tumor Growth *In Vivo*

**FIGURE 5:**
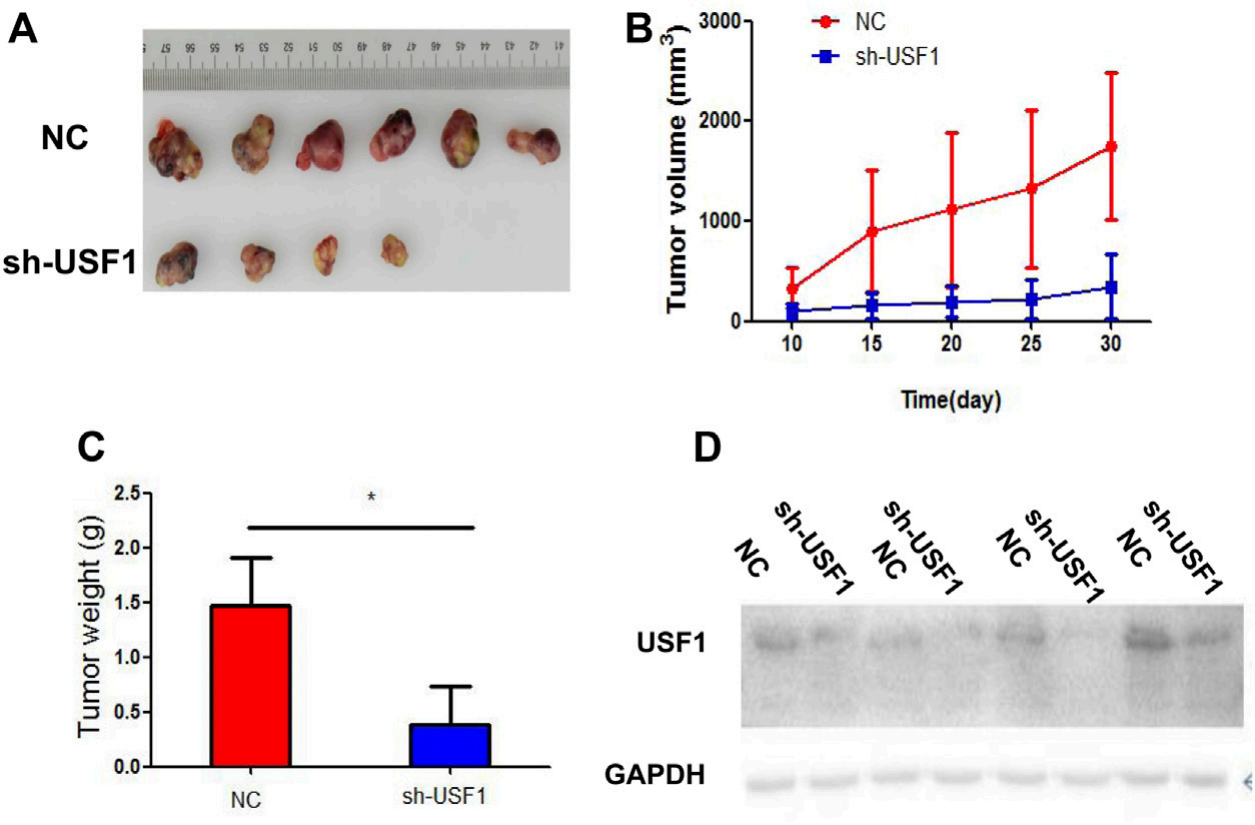
Investigation of USF1 on LUAD progression *in vivo*. **(A)** Measurement of the xenograft tumor size in different groups, *n* = 12; **(B)** The xenograft tumor growth curve; **(C)** The weight of the tumor xenograft; **(D)** Transfection efficiency, determined by WB. (Controls were designated as the NC group and represented mice transfected with sh-NC; while sh-USF1 group mice stood for those transfected with sh-USF1; *p < .05 means statistically significant difference when compared with NC group).

To further confirm the effect of USF1 on LUAD, xenografts mouse models were constructed by injection of H1299 cells into C57BL/6 mice. The shRNA specific against USF1 was used to treat mice *via* tail-vein injection. Thirty days later, tumor size in mice treated with USF1 shRNA was smaller than that in NC group of mice (Figure 5A). In line with this, tumor volume and weight of mice in USF1 shRNA group were lower than those in NC group (*p* < .05, Figures 5B,C). The expression changes of USF1 in tumor tissues following shRNA injection were confirmed by Western blotting analysis. It was shown that, the expression levels of USF1 were decreased in mice transfected with USF1 shRNA in comparison with the NC group (Figure 5D). These data showed that, knockdown of USF1 significantly inhibited the tumor growth of mouse models.

### DEGs in USF1- or UGT1A3-Silenced LUAD Cells

**FIGURE 6:**
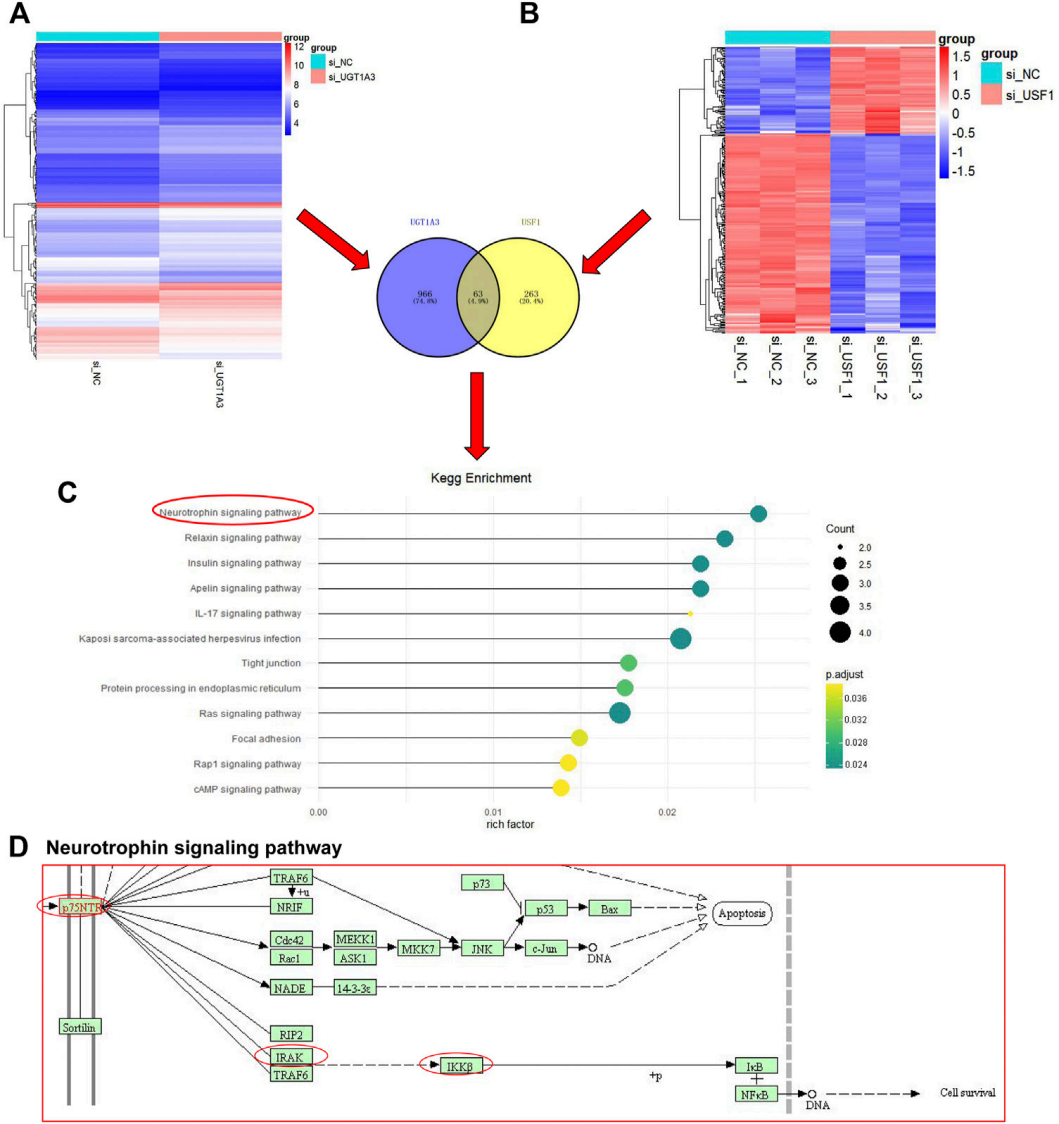
Identification and KEGG pathway enrichment analysis of DEGs. The si-UGT1A3, group cells were transfected with UGT1A3; **(A)** DEGs in cells knockdown of UGT1A3 determined by DNA micro-assay; **(B)** DEGs in cells knockdown of USF1 determined by DNA micro-assay; Venn diagram of the overlapping genes USF1 and UGT1A3; **(C)** KEGG pathways enriched by DEGs; **(D)** Pathway signature protein predicted by STRING.

Next, H1299 cells were transfected with shRNAs specific against UGT1A3 or USF1, and DEGs in cell was analyzed by DNA micro-assay. A total of 326 and 1,029 DEGs were found in cells transfected with UGT1A3 shRNA (Figure 6A) and USF1 siRNAs (Figure 6B), respectively. The Venn diagram showed that there are 63 overlapping genes. The KEGG pathway enrichment analysis indicated that these DEGs were significantly enriched in 12 pathways, of which neurotrophin signaling pathway ranked top one with the highest enrichment score (Figure 6C). The main signal elements involved are p75NTR, RIPK2, IRAK1, TRAF5, and IKKβ (Figure 6D). These results indicated that the neurotrophin signaling pathway in LUAD was activated by USF1 and UGT1A3.

### USF1 Knockdown Inhibited LUAD Cell Proliferation and Invasion *via* Inhibition of the Neurotrophin Signaling Pathway

**FIGURE7:**
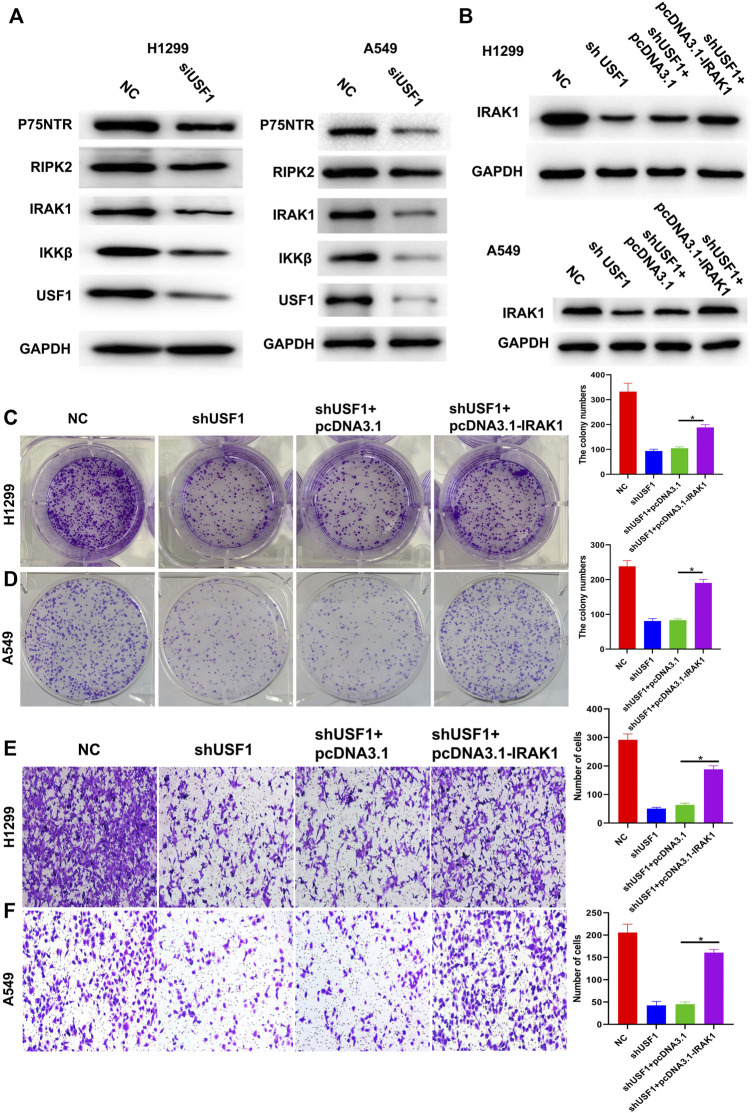
Knockdown of USF1 inhibited cells proliferation, migration and invasion *via* deregulation of the neurotrophin signaling pathway. **(A)** The protein expression of p75NTR, RIPK2, IRAK1, IKKβ and USF1, detected by WB in H1299 and A549 cells; **(B)** The levels of IRAK1 was detected by WB in H1299 and A549 cells; **(C,D)** Cells migration was detected by wound healing assay in H1299 and A549 cells; **(E,F)** Cells migration and invasion were detected by transwell assay in H1299 and A549 cells; (The sh-USF1, cells were transfected with sh-USF1; sh-USF1+pcDNA3.1, cells were transfected with sh-USF1 and pcDNA3.1; sh-USF1+ pcDNA3.1- IRAK1; *p < .05 means statistically significant difference when compared with sh-USF1+ pcDNA3.1 group).

Further studies aimed to reveal whether USF1 exerted its oncogenic role in LUAD *via* regulating neurotrophin signaling pathway. Western blotting results indicated that, the levels of p75NTR, RIPK2, IRAK1, IKKβ, and USF1 were downregulated in H1299 and A549 cells transfected with USF1 siRNA when compared with NC (Figure 7A). Besides, the expression levels of IRAK1 in H1299 and A549 cells were downregulated by transfection with USF1 shRNA, and upregulated by transfection with IRAK1 overexpression plasmid (Figure 7B). The colony formation of H1299 and A549 cells was inhibited by USF1 shRNA, and increased by IRAK1 overexpression plasmid (*p* < .05, Figures 7C,D). Additionally, the migration of H1299 and A549 cells was inhibited by USF1 shRNA, and increased by IRAK1 overexpression plasmid (*p* < .05, Figures 7E,F). These data showed that knockdown of USF1 inhibited LUAD cell proliferation and invasion *via* inhibition of neurotrophin signaling pathway.

## Discussion

LUAD originates from the bronchial mucosal epithelium and mucous glands (Chen et al., 2012). It can be asymptomatic in the early stages with various unspecific symptoms in the later stage. The incidence and mortality of LUAD are increasing every year (Zhang et al., 2019). Currently, there are no specific tumor markers for LUAD, and multiple indicators are used for joint detection to increase the detection rate (Chen et al., 2018). Therefore, at present, it is urgent to find novel molecular biomarkers for LUAD diagnosis, treatment and prognosis, and to provide explanations for the formation and progression of this type of tumor. TCGA database stores more than 20 kinds of cancer genome data, including mutations, copy number variations, mRNA expression, miRNA expression, methylation data, etc. which are widely used in cancer research (Blum et al., 2018).

UGT1A3 plays an important role in intestinal and liver drug metabolism, participating in the metabolism of a variety of cancers, thus promoting the resistance of tumor cells to chemotherapy drugs (Preston Pugh et al., 2014; Liu et al., 2015). In our previous study, we indicated that UGT1A3 was an important prognostic factor for LUAD, inhibiting UGT1A3 could significantly improve the efficacy of anti-tumor drugs (Wang et al., 2019). In this study, USF1 was found to be a transcriptional factor of UGT1A3. By analyzing TCGA database, USF1 was highly expressed in LUAD and its expression was associated with clinical pathologic characteristics of patients with LUAD, including tumor stage, nodal metastases, and poor survival rate. This finding suggested that USF1 directly binds to the promoter of UGT1A3 and contributes to its overexpression in LUAD.

The protein encoded by the USF1 gene regulates the expression of multiple genes which are related to glucose and lipid metabolism (Guo et al., 2020). Recent studies have found that USF1 is involved in regulating the development of multiple type of tumors, including lung cancer (Chen et al., 2021). For example, USF1 transcriptionally regulated GAS6-AS2 expression to promote the proliferation, migration and invasion of osteosarcoma cells (Wei et al., 2020). The transcription of HAS2-AS1 was activated by USF1, and the highly expressed HAS2-AS1 contributed to the migration, and invasion of glioma cells (Wang et al., 2020). USF1 activated WDFY3-AS2 expression to promote the progression of LUAD (Ren et al., 2020). Our data suggested that USF1 acted as a transcription factor for inducing UGT1A3 expression, and though which USF1 exerted its oncogenic roles in LUAD. Actually, the oncogenic effects of USF1 in lung cancer have been previously reported (Ren et al., 2020; Chen et al., 2021). However, we for the first time linked USF1 with UGT1A3 in the progression of LUAD.

DNA micro-assay can sequence hundreds of thousands or even millions of DNA sequences at the same time, and can comprehensively analyze the overall transcriptome and genome of a certain species, and then screen and analyze DEGs (Otero-Rey et al., 2004). By using DNA micro-assay, 63 overlapping DEGs were found in H1299 cells with USF1 and UGT1A3 knockdown, respectively. KEGG pathway enrichment analysis further indicated neurotrophin signaling pathway as a possible downstream signaling of USF1 in LUAD. Neurotrophins form a family of growth factors which play significant roles in neuronal development, such as survival, differentiation, axon outgrowth, and apoptosis (Rouigari et al., 2018). The biological effects of neurotrophins are mediated mainly *via* two receptors: the Trk family of receptor tyrosine kinases and the common p75NTR (Hempstead, 2014). Neurotrophins and their receptors are highly expressed in ovarian cancer (Davidson et al., 2001), breast cancer (Hondermarck, 2012), thyroid cancer (Faulkner et al., 2018), and lung cancer (Ricci et al., 2001). Moreover, neurotrophins and their receptors are proved to be involved in the progression of various cancers by stimulating cancer cell growth and dissemination (Griffin et al., 2018). Nerve growth factor (NGF), through its receptor p75NTR, promoted the migration of melanoma cells (Truzzi et al., 2008). NGF inhibited the growth of small-cell lung carcinoma cells and repressed tumorigenic potential in nude mice (Missale et al., 1998). It seems that NGF exerts both stimulatory and inhibitory effects on human cancers with overall effects determined by the ratio of TrkA to p75NGFR (Dang et al., 2006). In the present study, PPI network of KEGG pathway analysis showed P75NTR, RIPK2, IRAK1, TRAF5, and IKKβ as critical members involved in USF1-mediated downstream signaling, demonstrating the involvement of neurotrophin signaling pathway in LUAD.

IRAK1 is a serine/threonine-specific protein kinase that frequently expressed in solid tumors, including lung cancer (Pilarsky et al., 2004; Zhang et al., 2014). IRAK1 has been previously demonstrated to be critical in the activation of NGF-mediated NF-κB signaling (Mamidipudi et al., 2002; Mamidipudi et al., 2004). To be specific, NGF induces co-association of IRAK1 with PKC, and IRAK1 is recruited to P75NTR which further leads to the activation of NF-κB. In this study, same signaling pathway was observed in LUAD cells. In addition, *in vitro* experimental data suggested that, USF1 promoted the proliferation and invasion of LUAD cells possibly *via* regulating P75NTR/IRAK1 crosstalk mechanisms.

## Conclusion

Our study demonstrated that USF1 was highly expressed in LUAD in patients’ tissues, experimental cell lines, and mouse models. Knockdown of USF1 inhibited the viability, proliferation, migration, and invasion of LUAD cell lines, and reduced the tumorigenic potential in mouse models. Besides, USF1 directly bound to be promoter of UGT1A3 through which upregulated UGT1A3 expression and induced the activation of neurotrophin signaling pathway. The findings of this study provided a novel therapeutic target for LUAD.

## Data Availability

The original contributions presented in the study are included in the article/Supplementary Material, further inquiries can be directed to the corresponding author.
